# A High-Throughput Fluorescence-Based Assay System for Appetite-Regulating Gene and Drug Screening

**DOI:** 10.1371/journal.pone.0052549

**Published:** 2012-12-26

**Authors:** Yasuhito Shimada, Minoru Hirano, Yuhei Nishimura, Toshio Tanaka

**Affiliations:** 1 Department of Molecular and Cellular Pharmacology, Pharmacogenomics and Pharmacoinformatics, Mie University Graduate School of Medicine, Tsu, Mie, Japan; 2 Mie University Medical Zebrafish Research Center, Tsu, Mie, Japan; 3 Department of Bioinformatics, Mie University Life Science Research Center, Tsu, Mie, Japan; 4 Department of Omics Medicine, Mie University Industrial Technology Innovation Institute, Tsu, Mie, Japan; National University of Singapore, Singapore

## Abstract

The increasing number of people suffering from metabolic syndrome and obesity is becoming a serious problem not only in developed countries, but also in developing countries. However, there are few agents currently approved for the treatment of obesity. Those that are available are mainly appetite suppressants and gastrointestinal fat blockers. We have developed a simple and rapid method for the measurement of the feeding volume of *Danio rerio* (zebrafish). This assay can be used to screen appetite suppressants and enhancers. In this study, zebrafish were fed viable paramecia that were fluorescently-labeled, and feeding volume was measured using a 96-well microplate reader. Gene expression analysis of brain-derived neurotrophic factor (*bdnf*), knockdown of appetite-regulating genes (neuropeptide Y, preproinsulin, melanocortin 4 receptor, agouti related protein, and cannabinoid receptor 1), and the administration of clinical appetite suppressants (fluoxetine, sibutramine, mazindol, phentermine, and rimonabant) revealed the similarity among mechanisms regulating appetite in zebrafish and mammals. In combination with behavioral analysis, we were able to evaluate adverse effects on locomotor activities from gene knockdown and chemical treatments. In conclusion, we have developed an assay that uses zebrafish, which can be applied to high-throughput screening and target gene discovery for appetite suppressants and enhancers.

## Introduction

Most vertebrates can store a considerable amount of energy as fat for later use. This ability is currently responsible for major health risks facing humans worldwide. These risks include obesity and metabolic syndrome associated with cardiovascular disease. The predisposition to develop obesity can theoretically result from any pathological malfunction or lack of adaptation to changing environments [1]. The mechanism of feeding adaptation to obtain the correct amount of food is a response to the energy requirement of the body, amount of body fat, and blood glucose concentration. This is mediated by signaling molecules such as leptin and insulin [2]. Information is then transmitted to the central nervous system to regulate appetite [3]–[5]. In the short-term, such as during feeding, food consumption is regulated by several gut hormones, including cholecystokinin [6], peptides YY [7], oxyntomodulin [8], and neuropeptide Y (NPY) [9], [10]. These multi-organ mechanisms of appetite regulation make it difficult to discover appetite-regulating targets and agents, resulting in very few FDA approved drugs for treating obesity and metabolic syndrome. In drug discovery, high-throughput screening (HTS) is the most efficient method to detect appetite suppressants and promoters, because conventional rodent-feeding quantification systems require intensive labor, time, and many animals [11]. This makes it almost impossible to find lead compounds from libraries of chemicals with varying concentrations with several genetic interventions. Thus, there is an urgent need for new feeding behavior assays using alternative animal models with HTS capacity. Over the last decade, to overcome the bottlenecks surrounding throughput and complexity in whole-animal testing, phenotype-based screening of *Danio rerio* (zebrafish) has emerged as a powerful tool for identifying small bioactive compounds. The use of zebrafish has many advantages over traditional models, as they are simple to maintain, have a high reproductive capacity, and develop rapidly. Additionally, fluorescence-based zebrafish screening using reporter-based transgenes allows visualization through their transparent body walls, allowing for HTS. Compared with other model organisms used in drug screening (yeast, nematodes, and fruit flies), zebrafish possess many evolutionary and morphological similarities to humans [12]. Also, various models of human disease have been established in zebrafish [13]–[15] for genetic disorders [16] and diet-induced obesity [17], [18]. For appetite regulation, zebrafish npy [10], agouti-related protein [16], melatonin 4 receptor, and melatonin [19] are well conserved in appetite or metabolic regulation. These features make this species a versatile tool for pre-clinical drug discovery and toxicological investigation. The aim of our study was to create a simple *in vivo* HTS system to evaluate feeding volume. Here, we present a novel feeding evaluation assay using zebrafish larvae with fluorescently-labeled live bait, paramecia. Living paramecium is one of the most suitable foods for zebrafish and other small fish larvae because of its nutritional components and appetite induction in young fish [20], [21]. In addition, using knockdown experiments and known human appetite suppressants, we demonstrated that the genes and drug responses involved in appetite regulation are very similar in zebrafish and mammals.

## Materials and Methods

### Ethics Statement

All animal experiments were conducted according to the ‘Act on Welfare and Management of Animals’ (Ministry of Environment of Japan) and complied with international guidelines. Ethics approval from the local Institutional Animal Care and Use Committee was not sought, since this law does not mandate protection of fish. After the experiments, the fish were sacrificed by anesthetic overdose.

### Chemicals

Fluoxetine hydrochloride and phentermine hydrochloride were purchased from Sigma-Aldrich (St Louis, MO, USA). Sibutramine hydrochloride monohydrate was purchased from Wako Pure Chemicals (Tokyo, Japan). Mazindol was purchased from Tokyo Kasei (Tokyo, Japan). Rimonabant hydrochloride was purchased from Santa Cruz Biotechnology (Santa Cruz, CA, USA). Stock solutions (10 mM) of the drugs were prepared in dimethyl sulfoxide (Sigma-Aldrich). The anesthetic used was 3-aminobenzoic acid ethyl ester (tricaine, MS-222), which was also purchased from Sigma-Aldrich. For anesthesia, 1.6 mg/ml tricaine was dissolved in E3 medium (5 mM NaCl, 0.17 mM KCl, 0.33 mM CaCl_2_, and 0.33 mM MgSO_4_) supplemented with 5 mM HEPES and adjusted to pH 7.0. This was a 10× anesthetic solution for use in young zebrafish.

### Zebrafish Breeding

Zebrafish (AB strain) were obtained from the Zebrafish International Resource Center of the University of Oregon. Fish were maintained on a 14∶10 h light:dark cycle and bred in our laboratory according to standard conditions [21]. Embryos obtained from natural mating were kept in E3 medium at 28°C.

### Preparation of Fluorescently-labeled Paramecia

Paramecia were a kind gift from Dr. K. Araki (National Research Institute of Aquaculture, Mie, Japan). Paramecia were cultured in medium consisting of one tablet of dry yeast (EBIOS, Asahi Food and Healthcare, Tokyo, Japan), and four grains of wheat germ dissolved in 1 l of distilled water. At the appropriate density, paramecia culture medium was transferred into a shading bottle, whose upper part was transparent. Since paramecia are attracted to light, they were collected from the culture medium close to the surface. The collected paramecia medium was filtered through a coarse mesh (112 µm) filter (PP-112n; Kyoshin Riko, Tokyo, Japan) to remove debris, and then filtered through a fine mesh (20 µm) filter (PP-20n; Kyoshin Riko) to obtain pure paramecia. The paramecia were then resuspended in 50 ml of distilled water. To concentrate the purified paramecia, the suspension was centrifuged at 3,000 rpm with a Kubota 8800 centrifuge (Kubota, Tokyo, Japan) for 5 min and resuspended again in 1 ml of distilled water. Paramecia were quantified by measuring the optical density (500 nm) of a 10-fold dilution using a spectrophotometer (Beckman DU600 spectrophotometer, Beckman Instruments, Fullerton, CA, USA). The following calculation was used to determine the amount of paramecia: paramecia (P) = optical density of the 10-fold dilution at 500 nm (OD_500_) × 10 × volume (V) in µl. For fluorescent labeling of paramecia, we used the lipophilic tracer 4-(4-(didecylamino)styryl)-*N*-methylpyridinium iodide (4-Di-10-ASP; Invitrogen, Carlsbad, CA, USA), which is usually used for live-cell staining in neurons [22], hair cells [23], and cancer cells for xenotransplantation [24]. Almost 1000 P of paramecia were suspended in 1 ml of distilled water, then 25 µg/ml 4-Di-10-ASP was added. The suspension was gently mixed by inverting the tube. After 1 h of staining, paramecia were washed, by centrifuging, to remove residual 4-Di-10-ASP.

### Quantification of Paramecia Intake

In zebrafish, the rostral digestive tract is fully functional by 3 days post-fertilization (dpf), when the lumen of the posterior pharynx is visible and the mouth is open, and posteriorly, when the anus is open at 4 dpf [25]. Thus, we conducted labeled paramecia feeding from 5 dpf until 10 dpf because of optimal body wall transparency and size for the 96-well plates. We conducted paramecia feeding in 6-well plates to allow for free swimming of zebrafish. At 12 h before feeding with paramecia, 15 larvae per condition were transferred into 6-well plates containing 3 ml of E3 medium. A total of 1000 P of fluorescent paramecia were added into each well. Two hours after feeding, larvae were anesthetized. After two washes with fresh E3 medium to remove residual paramecia, each larva was transferred into a 96-well round bottom black plate (3792; Corning, Lowell, MA, USA) together with 20 µl of anesthetic solution. The intra-abdominal fluorescent signal was measured using the Victor2 fluorescent plate reader (PerkinElmer, Boston, MA, USA), in well scan mode (10 × 10 multipoint/well, 0.1 s/point, 5 repeats) using a fluorescein filter set (excitation wavelength 485 nm, emission wavelength 535 nm). The sum of each point was defined as proportional to the amount of paramecia fed to each zebrafish.

### Swimming Behavioral Analyses

To determine whether gene knockdown and drug treatment negatively affected zebrafish neurobehavioral function, swimming behavior was analyzed as previously described [26], with some modifications. One hour prior to evaluation, a fish was removed from each experimental unit and placed in a well with 50 µl of E3 medium in a 96-well clear bottom plate for acclimatization. The fish were then video recorded for 15 min, and the resulting footage evaluated for swimming distance using the EthoVision XT system (version 8.0; Noldus Information Technology, Wageningen, The Netherlands).

### Drug Treatment

The following drugs were used in treatments: fluoxetine, a selective serotonin reuptake inhibitor; sibutramine, a serotonin and noradrenalin reuptake inhibitor; mazindol, a catecholamine reuptake inhibitor; phentermine, a catecholamine releaser; and rimonabant, a cannabinoid type 1 receptor antagonist. Drugs were added to fish in 6-well plates for 12 h before feeding with paramecia.

### Gene Knockdown and mRNA Rescue

For targeted gene knockdown, morpholino antisense oligonucleotides (MO) were synthesized by GeneTools (Philomath, OR, USA). The MO sequences are shown in Table 1. Each MO was injected into single-cell-stage embryos at a concentration of approximately 1 ng per embryo, in a solution of sterile water and 0.1% phenol red (Sigma-Aldrich). For the negative control groups, the standard MO (human β-globin mutant sequence; GeneTools) was injected into embryos. The MO solution was mixed with 50 ng/µl of pXI-GFP plasmid [27], and used as a marker for MO introduction. For mRNA synthesis, we amplified the open reading frames of *insa* and *npy* by PCR and inserted *Eco*RI/*Sma*I and *Xho*I/*Sal*I sites, respectively, into the pTNT vector (Promega, Madison, WI, USA) using a Mighty Mix DNA Ligation Kit (Takara Bio, Ohtsu, Japan). All restriction enzymes were purchased from New England Biolabs (Beverly, MA, USA). The primer sequences are shown in Table 2. The mRNA corresponding to *insa* and *npy* were then synthesized using mMessage Machine (Ambion Life Technologies, Grand Island, NY, USA). To rescue *insa* and *npy* MO-induced appetite suppression, 200 pg of synthesized mRNA per embryo were co-injected with each MO.

**Table 1:** Morpholino oligonucleotides used in the present study.

Target gene	Locus ID	MO name	Sequence (5' - 3')	Type of inhibition
preproinsulin a	NM_131056	insa MO	GCA CCA GCC TGA AGC CAC ACT GCC A	Translational
preproinsulin b	NM_001039064	insb MO	GTG GAC ACA GAC TGA GGA CAT GCA C	Translational
neuropeptide y	NM_131074	npy MO	CCA CAT CTT CAT GTT TGG ATT CAT C	Translational
brain-derived neurotrophic factor	NM_131595	bdnf MO	GCT GAA TGG TCT CCT TTA CGA CTG G	Translational
melanocortin 4 receptor	NM_173278	mc4r MO1	ATGATGATGTGAGGTGTTCATTTCC	Translational
		mc4r MO2	TGATCCCTGTTTAAGTCAGTCTGGT	Translational
agouti related protein homolog	XM_001923904	agrp MO1	GGCAGTGAGTCTATGATGTACAAAA	Splicing
		agrp MO2	GATAAATGCCAGTAAACCACCTCAT	Splicing
cannabinoid receptor 1	NM_212820	cnr1 MO	GTGCTATCAACAACATACCTTTGTG	Splicing

### Total RNA Extraction

Larvae were exposed to fluoxetine and sibutramine in 6-well plates at 5 dpf and sacrificed 12 h after deep anesthetization. For total RNA extraction from the brain, the epidermis, gills, swim bladder and intestinal tract of each larva were removed. Larvae were dissected so that individual brain regions could be obtained [28]. Dissected tissues were immersed in RNAlater (Life Technologies) and kept at 4°C overnight. Tissues were then placed in RLT buffer (Qiagen, Hilden, Germany) and homogenized using an MM300 Mixer Mill (Retsch, Haan, Germany) at 30 Hz for 2 min. Total RNA was extracted from tissues and treated with DNaseI from an RNeasy Micro Kit (Qiagen) according to the manufacturer’s instructions. For total RNA extraction from the whole body, larvae were homogenized in RLT buffer using the MM300 Mixer Mill and total RNA purified using the RNeasy Mini Kit.

### cDNA Synthesis, Polymerase Chain Reaction (PCR) and Quantitative PCR (qPCR)

First strand cDNA was prepared with 200 ng of total RNA using the Super Script III First-strand System with random primers (Invitrogen). All qPCR assays were performed using Power SYBR Green Master Mix (Applied Biosystems, Foster City, CA, USA) in triplicate according to the manufacturer’s instructions. Data were normalized to zebrafish beta-actin 1 (*actb1*). PCR products were separated by agarose (2%) gel electrophoresis, stained with ethidium bromide and visualized using a UVP BioDoc-It System (Bioimaging systems, Upland, CA, USA). Primers were synthesized by Invitrogen and their sequences are shown in Table 2.

**Table 2:** Primer sequences used in the present study.

Gene symbol	Gene name	Locus ID	Purpose	Forward primer sequence (5'-3')	Reverse primer sequence (5'-3')
insa	preproinsulin a	NM_131056	Cloning	TCGAATTC ATGGCAGTGTGGCTTCAGGC	ACGTCTTGATGACATTGACTGGGCCCTA
npy	neuropeptide y	NM_131074	Cloning	GTCCTCGAG ATGAATCCAAACATGAAGAT	TCCTAGTAAACCGTACCACTGTCGACTA
			QPCR	GACTCTCACAGAAGGGTATCC	GGT TGATGTAGTGTCTTAGTGCTG
actb1	actin, beta 1	NM_131031	QPCR	CTCTTCCAGCCTTCCTTCCT	CACCGATCCAGACGGAGTAT
bdnf	brain-derivedneurotrophic factor	NM_131595	QPCR	ATAGTAACGAACAGGATGG	GCTCAGTCATGGGAGTCC
slc6a4a	solute carrier family 6,member 4A	NM_001039972	QPCR	CATCTATGCTGAGGCTATTG	AAGAATATGATGGCGAAG
htr1aa	serotonin receptortype 1aa	NM_001123321	QPCR	ATGAGGATGAGCGGGATGTAG	CAATCAGCCAGGACCACG
htr1ab	serotonin receptortype 1ab	NM_001145766	QPCR	CTGTGTCGCCTGCACTTTTC	TGATCTCCAAAGACTCGCCG
agrp	agouti related protein	XM_001923904	PCR for MO inhibition	GGCTGGTTTTTGGTGAATGT	TCGTTTTTGCAGGTGTTGTC
cn1r	cannabinoid receptor 1	NM_212820	PCR for MO inhibition	ACGTCACAGAAGAGCCTGGT	GGCGATTTTCACAGTGGTCT
pTNT	Expression vector pTNT	AF479322	Cloning	AAGGCTAGAGTACTTAATACGA	GTCCATTCGCCATTCAGG

Underline indicates the restriction enzyme recognition sites.

### Western Blot

Larvae (100) at 6 dpf were homogenized in 250 µl of T-PER Tissue Protein Extraction Reagent (Pierce, Rockford, IL, USA) containing Halt Proteinase Inhibitor Cocktail (Pierce) and 0.5 mM EDTA. Homogenization was performed using an MM300 Mixer Mill (Retsch) at 30 Hz for 2 min at 4°C. The homogenate was centrifuged (13,000 rpm, 20 min, 4°C) to remove debris, and the supernatant collected for further analysis. Protein concentration was determined using a Bradford Ultra Protein Assay Kit (Expendeon, Cambridge, UK). Protein samples (30 µg) were heated to 70°C for 10 min and separated on 4–12% Bis-Tris gels using a NuPAGE SDS-PAGE Gel System (Invitrogen). Separated proteins were then transferred to polyvinylidene difluoride (PVDF) membranes (iBlot PVDF; Invitrogen) and blocked in PVDF Blocking Reagent (Toyobo, Osaka, Japan). Following blocking, blots were incubated in Can Get Signal Immunoreaction Enhancer Solution 1 (Toyobo), containing primary antibody, overnight at 4°C, followed by three 10-min washes with phosphate-buffered saline (PBS) containing Tween 20 (PBST). Polyclonal guinea-pig anti-porcine insulin (1∶1000 dilution; Dako Cytomation, Glostrup, Denmark), anti-neuropeptide Y (1∶1000; ImmunoStar, Hudson, WI, USA) and anti-GAPDH (1∶2000; Abcam, Cambridge, MA, USA) antibodies were used. The washed membrane was incubated in Can Get Signal Immunoreaction Enhancer Solution 2 (Toyobo) containing goat peroxidase-conjugated anti-rabbit IgG antibody (1∶1000; Abcam) for 1 h at 25°C. After three washes with PBS, the bound antibodies were detected by chemiluminescence (ECL; GE Healthcare, Milwaukee, WI, USA). The images were obtained with LAS-4000 (Fujifilm, Tokyo, Japan).

### Imaging

Larvae were mounted on a glass slide in 3% methylcellulose containing 0.16 mg/ml tricaine for immobilization. Images were captured using a MZ16F stereoscopic microscope (Leica Microsystems, Wetzlar, Germany) equipped with a DP71 digital camera (Olympus, Tokyo, Japan). For the imaging of 4-Di-10-ASP-labeled paramecia, a GFP2 filter was used (exposure time: 100 ms). Merged images were created using Adobe Photoshop software version 5.5 (Adobe Systems, Seattle, WA, USA).

### Statistics

Data are expressed as mean ± the standard error of the mean (SEM). Differences between the two groups were compared using Student’s *t*-test. For multiple comparisons, a one-way ANOVA followed by Dunnett’s test for multiple comparisons was used. A P-value less than 0.05 was considered statistically significant.

## Results

### Zebrafish Feeding Assay using Fluorescently-labeled Paramecia

**Figure 1:**
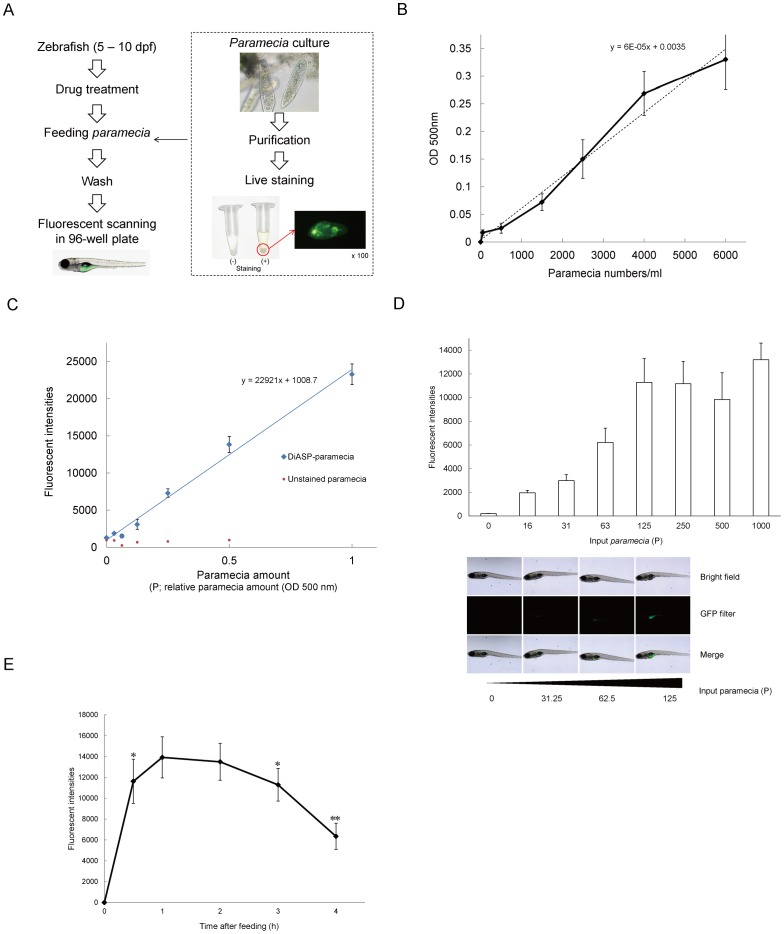
Zebrafish feeding assay using 4-Di-10-ASP labeled paramecia. (A) Schematic representation of the feeding assay. (B) The actual numbers of paramecia correlated with optical density at 500 nm (OD_500_). (C) Correlation between the relative amount of paramecia (P) and fluorescent intensity. (D) Fluorescent intensities of ingested paramecia in zebrafish. The fluorescent intensities of paramecia in zebrafish abdomen increased independently to the numbers of introduced-paramecia. All values are mean ± SEM, *n* = 15. (E) Fluorescent intensities after free-feeding of 4-10-Di-ASP-labeled paramecia. Fluorescent intensities of ingested paramecia were at maximum levels 1–3 h after feeding. All values are mean ± SEM, *n* = 15, *P<0.05, **P<0.01 compared with 1 h after feeding.

A schematic representation of the zebrafish feeding assay is summarized in Figure 1A. The numbers of paramecia in distilled water correlated to the absorbance at 500 nm (Fig. 1B), therefore we were able to estimate how many paramecia were present in each experiment. The lipophilic tracer 4-Di-10-ASP, stained paramecia for at least 3 days without any leakage from their bodies, and had no effect on morbidity and motility. After 4-Di-10-ASP labeling, paramecia levels were directly proportional to fluorescent intensity (Fig. 1C). Fluorescent signals from ingested paramecia in the abdominal area were measured. It was difficult to determine the exact location of the gut for each larva, therefore fluorescent intensity was measured at multiple points. The relationship between the amount of introduced and ingested paramecia can be clearly seen (Fig. 1D). Two hours after feeding, fluorescent intensities increased, depending on the amount of paramecia. In addition, free feeding of the maximum volume of paramecia (1000 P) showed that the feeding volume peaked 1 h after feeding started (Fig. 1E). At 0.5 and 3 h after feeding had started, the fluorescent intensity was significantly less than that at 1 h (*P*<0.05). At 4 h, fluorescent intensity was further reduced (*P*<0.01). There was no significant difference in fluorescent intensity between 1 and 2 h after feeding. Thus, these data suggested that the peak of feeding volume existed during 1–3 h after feeding. Following these experiments, the feeding time was set at 2 h, which ensured enough time for maximum paramecia intake.

### Gene Expression Related to Regulation of Appetite in Zebrafish and Humans

**Figure 2:**
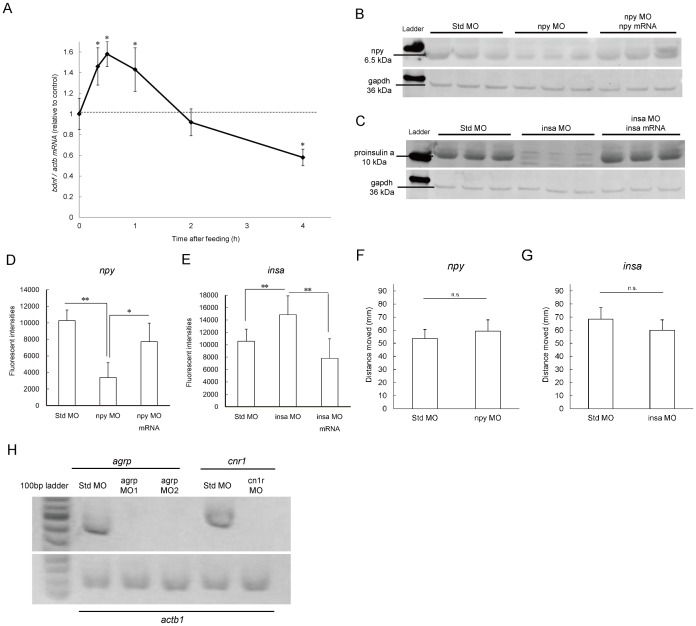
Gene expression and the effect of knocking down genes associated with appetite regulation. (A) Analysis by qPCR of brain-derived neurotrophic factor mRNA (*bdnf*) during paramecia feeding, *n* = 5, *P<0.05. (B and C) Western blots showing embryonic injection of neuropeptide Y (*npy*) MO (B) and preproinsulin a (*insa*) MO. (D) Embryonic injection of *npy* MO and *npy* mRNA. Orexigenic gene knockdown decreased feeding volume. (E) *insa* MO and *insa* mRNA, orexigenic gene knockdown increased feeding volume at 7 dpf. *n* = 15, *P<0.05. All values are mean ± SEM. (F and G) Knockdown of *npy* (F) and *insa* (G) affected locomotor activities at 5 dpf. *n* = 16, *P<0.05. All values are mean ± SEM. (H) Detection of gene knockdown for splicing MOs in 2% (w/v) agarose gels stained with ethidium bromide.

To test whether the zebrafish feeding assay could be used to explore appetite-regulation in mammalian species, we conducted gene expression analysis and knockdown studies. Brain-derived neurotrophic factor (BDNF) is one of the most important anorexigenic factors in the mammalian hypothalamus and regulates feeding behavior to stop feeding [29]. In zebrafish, *bdnf* mRNA expression was significantly increased (*P*<0.05) within 30 min of the initiation of feeding (Fig. 2A). Feeding-induced *bdnf* expression stimulates the satiety center and is a common mechanism of appetite regulation in vertebrates. We next conducted gene knockdown experiments using MO with appetite-regulating genes and mRNA rescue experiments. The knockdown efficacies of *npy* MO and *insa* MO were confirmed by western blotting (Fig. 2B and C). The knockdown of *npy*, a major orexigenic hormone also found in zebrafish [10], decreased food intake by 67% compared with the control-MO injected group at 7 dpf (*P*<0.05; Fig. 2D). Conversely, knockdown of *insa*, an ortholog of the human precursor of insulin, preproinsulin [30], increased food intake by 41% (Fig. 2E; *P*<0.05). The effects on appetite modulation induced by npy and insa MO were rescued by each mRNA co-injection (Fig. 2D and E). Knockdown of *npy* and *insa* had no effect on the distance of spontaneous swimming (Fig. 2F and G). Zebrafish have another insulin paralog, preproinsulin b (*insb*), derived from gene duplication [30]. *insb* expression peaked at 1 dpf, and *insb* knockdown caused severe abnormalities, high mortality, and virtually eradicated the population by 5 dpf. In addition, *bdnf* knockdown caused severe pericardial swelling and reduced locomotor activity similar to a previous study [31]. We conducted melanocortin 4 receptor (mc4r), agouti-related protein (agrp), and cannabinoid receptor 1 (cnr1) knockdown (Table 3). The mc4r gene is a major orexigenic gene in zebrafish appetite regulation systems [33]. And the mc4r MO injection increased food intake (*P*<0.05). Regarding agrp, a strong inhibitor of melanocortin systems in fish and mammals, the agrp MO significantly decreased (*P*<0.05) food intake and locomotor activity. The cnr1 MO decreased locomotor activity (*P*<0.01) with suppression of feeding behavior. The knockdown efficacies of these splicing inhibitor MOs (agrp MO1, 2 and cnr1 MO) were confirmed by PCR (Fig. 2H).

**Table 3:** Effects of morpholinos on food intake and locomotor activity.

Target gene	MO name	Food intake (ratio to Std MO)	Distance of spontaneous swimming (ratio to Std MO)
npy	npy MO	0.33±0.17*	1.12±0.16
insa	insa MO	1.41±0.29*	0.88±0.12
insb	insb MO	(lethal)	(lethal)
bdnf	bdnf MO	nd	0.01±0.00**
mc4r	mcr4 MO1	1.38±0.32*	0.93±0.15
	mcr4 MO2	1.45±0.23*	1.05±0.21
agrp	agrp MO1	0.64±0.17*	0.42±0.20*
	agrp MO2	0.72±0.13*	0.58±0.21*
cnr1	cnr1 MO	0.27±0.16**	0.19±0.11**

*n* = 16, *P<0.05, **P<0.01. All values are mean ± SEM. nd; not detected. Red color indicates increase and green color indicates decrease in each parameter.

### Appetite Suppressant Treatment in Zebrafish

**Figure 3:**
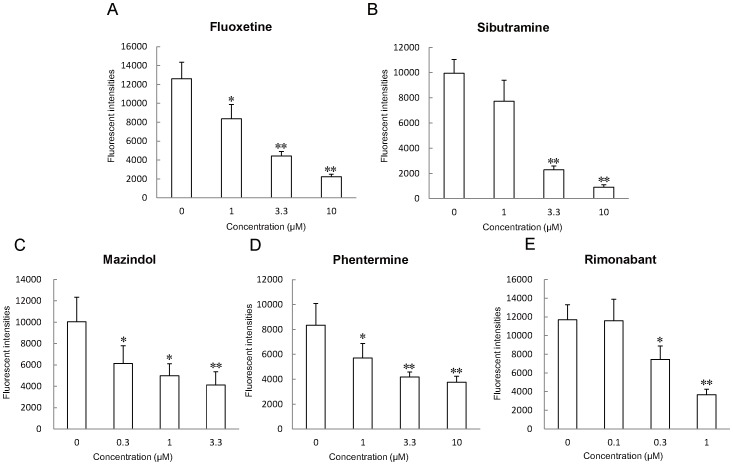
Effect of appetite suppressants on feeding volume. Effects of appetite-regulating drugs that are used clinically in humans affected zebrafish larvae. (A) Fluoxetine, (B) Sibutramine, (C) Mazindol, (D) Phentermine, and (E) Rimonabant treatment decreased feeding volume in young zebrafish (7 dpf). All values are mean ± SEM, *n* = 16, *P<0.05, **P<0.01.

**Figure 4:**
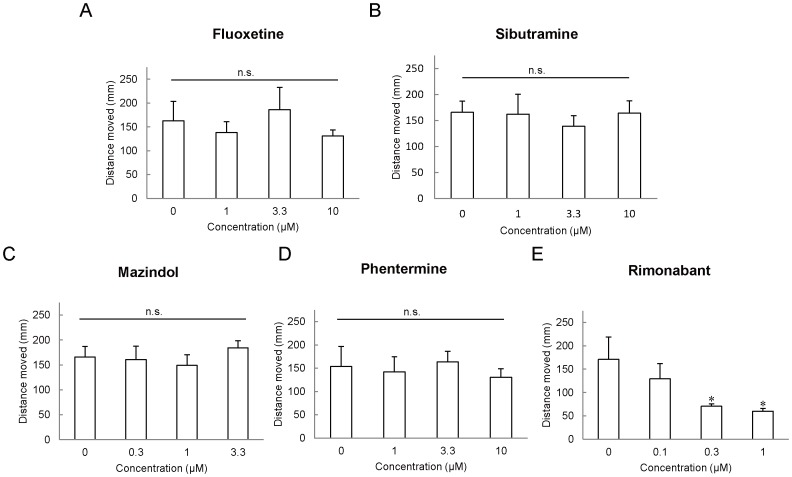
Effect of appetite suppressants on locomotor activities. The effects of appetite-regulating drug treatment on spontaneous swimming (15 min) were measured in young zebrafish. (A) Fluoxetine, (B) Sibutramine, (C) Mazindol, (D) Phentermine, and (E) Rimonabant. All values are mean ± SEM, *n* = 16, *P<0.01.

We validated whether appetite suppressants decrease zebrafish larvae feeding in the assays we developed. The feeding evaluation results after the administration of fluoxetine (Fig. 3A), sibutramine (Fig. 3B), mazindol (Fig. 3C), phentermine (Fig. 3D), and rimonabant (Fig. 3E) can be clearly seen. Overnight treatment with these drugs suppressed food intake in a dose-dependent manner despite different anorexic mechanisms of action for each of the drugs. In addition, the drugs had no effect on morbidity or motility during the experiments. Figure 4 shows the distance of spontaneous swimming. Of the drugs used in this study, only rimonabant at 0.3 and 1 µM significantly decreased (*P*<0.05) the distance moved compared with controls (Fig. 4E).

### Gene Expression Analysis Following Fluoxetine and Sibutramine Treatment

**Figure 5:**
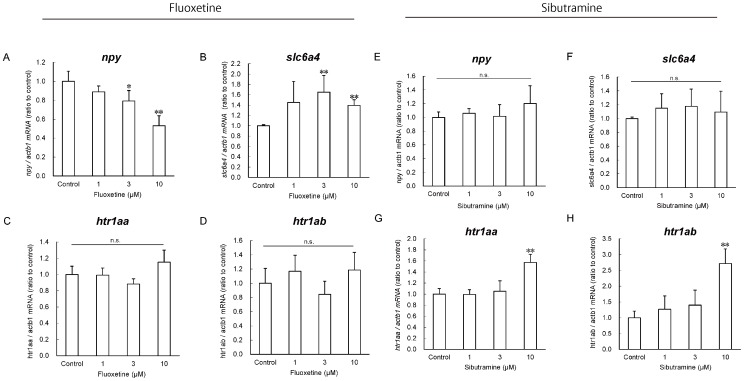
Gene expression analyses following fluoxetine and sibutramine treatment. Downstream genes of appetite regulation in young zebrafish brains were measured after treatment with fluoxetine (A–D) and sibutramine (E–H). (A and E) *npy*, (B and F) *slc6a4*, (C and G) *htr1aa*, and (D and H) *htr1ab*. All values are mean ± SEM, *n* = 4, *P<0.05, **P<0.01.

We performed gene expression analyses on fluoxetine- and sibutramine-treated zebrafish. Fluoxetine treatment decreased *npy* expression in a dose-dependent manner, but increased *serotonin transporter* (*slc6a4a*) expression (*P*<0.01; Fig. 5A and 5B). Sibutramine treatment (10 µM) induced *serotonin receptor type 1aa* (*ht1aa*) and *1ab* (*ht1ab*) expression (*P*<0.01; Fig. 5G and Fig. 5H).

## Discussion

### *In vivo* Appetite-HTS System using Zebrafish

Several zebrafish-based behavioral assays for scototaxis (anxiety-like behavior in fish) [33], thigmotaxis [34], and social interaction [35] have been developed, and allow the evaluation of neurotransmitter systems for target genes and drug discovery [36]. For feeding behavior assays, prey capture tracking techniques using larvae [37], [38], measuring the volume of food consumed [32], and monitoring *Artemia* numbers [18] using adult zebrafish have been reported. The aim of the current study was to create a simple *in vivo* HTS system to evaluate feeding volume. We selected zebrafish larvae because they can be used in a 96-well format. In combination with our fluorescence-based feeding assay and other conventional neurobehavioral analyses, we were able to evaluate potential pharmacological and toxicological targets of feeding behavior using HTS.

### Conservation of Appetite-regulating Gene Function between in Humans and Zebrafish

Previous studies have shown conservation of gene function in several neurobehavioral responses against psychotropic drugs, and for neurobehavioral toxicities between humans and zebrafish [39], [40]. In this study, we demonstrated, using gene knockdown and appetite suppressant treatments, that the major appetite regulatory pathways of mammals, including those for BDNF, NPY, insulin, 5-HT, melanocortin and central adrenergic signaling, were highly conserved in zebrafish.

Insulin suppresses feeding behavior through its direct action on the hypothalamic satiety center, independently of the reduction of blood glucose levels in mammals [41], [42]. Zebrafish insulin gene knockdown increased feeding volume, suggesting functional similarities of zebrafish and human insulin signaling on appetite regulation in the central nervous system. In addition, appetite regulation by insulin signaling in mammals occurs via insulin receptors and insulin receptor substrate 2 (IRS-2) in the hypothalamus [43]. While irs-2 has not been identified in zebrafish yet, insulin receptors a and b are expressed in the zebrafish brain and regulate metabolic homeostasis along with ghrelin [44], suggesting that insulin signaling also regulates appetite in the zebrafish central nervous systems. The melanocortin system is also important in energy metabolism and for the feeding behavior of zebrafish [45], [46]. The mc4r is a major regulator of anorexigenic response and energy homeostasis in zebrafish [47] and mammals [48]. mc4r MO-zebrafish were hyperphasic and locomotor activity was not inhibited. Overexpression of agrp in zebrafish larvae resulted in obesity and adipocyte hypertrophy [16], while agrp knockdown resulted in decreased food intake with locomotor inhibition (Table 3). We did not observe shortening of body length following agrp knockdown at 6 dpf larvae, as previously reported [47]. It is possible that suppression of somatic development induced by the agrp MO can inhibit feeding behavior independent of the activation of melanocortin signaling.

The endcannabinoid receptor cnr1, mediates the psychoactive effect of marijuana and addiction [49]. It is also expressed in the periventricular hypothalamus of zebrafish [50]. This possibly suggests that cnr1 in zebrafish may share the same function in appetite regulation, and could be a target for rimonabant [51], [52]. Knockdown of cnr1 decreased the spontaneous locomotion (Table 3). The cnr1 MO probably inhibits normal axonal growth and fasciculation during brain development [53], and suppresses locomotor activity. This corresponds with the results of CNR1 antagonism in neurobehavioral disorders, such as Parkinson’s disease [54] and our result of rimonabant treatment (Fig. 3E and4E).

### Anorexic Drug Response in Zebrafish

Zebrafish exhibited significant suppression of their appetites in response to anorexic drugs. Fluoxetine (Fig. 3A) is a selective serotonin reuptake inhibitor (SSRI) used for the treatment of clinical depression, anxiety disorder, obsessive-compulsive disorder, and bulimia. SSRIs are potent and highly selective inhibitors of the transporter enzyme for serotonin (5-HT) reuptake at the presynaptic membrane, causing an increase in 5-HT concentrations at postsynaptic receptor sites [55]. Serotonin transporter genes are also conserved in zebrafish [56], [57]. It is well known that 5-HT plays an inhibitory role in the control of ingestive behavior, and that the administration of fluoxetine suppresses food intake and weight gain under free-feeding and food-restricted conditions [58]. In addition, it has been shown that fluoxetine reduces mRNA expression levels of *Npy* in rat brain [59]. Feeding behavior is also regulated by *npy* in zebrafish (Fig. 2D). The expression of *npy* in brain regions following fluoxetine treatment decreased in a dose-dependent manner (Fig. 5A), demonstrating that mechanisms regulating appetite are highly preserved between zebrafish and mammals. Serotonin transporter (*slc6a4*) mRNA was induced by fluoxetine treatment (Fig. 5B). In a previous study, sub-chronic administration of fluoxetine in rodents increased *Slc6a4* expression in the midbrain raphe [60], indicating a similarity in serotonergic appetite regulatory mechanisms in vertebrates. However, the time-course for fluoxetine pharmacodynamics in zebrafish is shorter than that in mammals. The expression of serotonin receptor type 1aa (*htr1aa*) and 1ab (*htr1ab*) were not altered by fluoxetine treatment, consistent with previous studies [61]. Sibutramine, a central-acting serotonin-norepinephrine reuptake inhibitor [62], also reduces food intake in zebrafish (Fig. 3B). In contrast to fluoxetine treatment, sibutramine did not result in a decrease in *npy* expression in brain regions (Fig. 5E). This result was also consistent with a previous study [63]. A high dose of sibutramine (10 µM) induced *htr1aa* and *htr1ab* expression, suggesting hypersensitivity and activation of 5-HT signaling, possibly increasing the anorexic effects of sibutramine. The adrenergic system of zebrafish has been well characterized by many researchers, and α2a-adrenoceptors have been shown to be expressed in the hypothalamus [64]. These findings suggest the involvement of adrenergic signaling in the satiety center of the zebrafish brain, similar to that seen in mammals [65]. Mazindol, a reuptake inhibitor of norepinephrine and phentermine (a releaser of norepinephrine), acts on the hypothalamus to stimulate the adrenal glands to increase norepinephrine, subsequently reducing hunger in zebrafish (Fig. 3C and D).

### Appetite-HTS with Evaluation of Locomotor Activities

Appetite suppressants exhibit many severe central nervous system side effects including depression, agitation, and anxiety [66], which have sometimes been reflected in locomotor activities in animal models. Thus, there is a need for the evaluation of appetite with, at the very least, locomotor activities under similar conditions to that of animal experiments. In the current study, rimonabant significantly suppressed appetite volume (Fig. 3E) and locomotor activities (Fig. 4E), which was consistent with cnr1 knockdown (Table 3). In addition to appetite suppression, rimonabant is associated with several adverse psychiatric events including depression and anxiety in humans [67], [68]. Rimonabant-induced suppression of locomotor activities in zebrafish likely reflects the clinical situation. In addition, it has been reported that serotonergic stimulation decreases locomotor activity in zebrafish [28], however we did not observe this in the current study. The reason for this difference is likely because of the shorter exposure time in this study compared with that in the previous study (10 µM for 12 h vs. 10 µM for 24 h). An intravenous injection of fluoxetine was not associated with significant effects on locomotor activity in chinook salmon [69]. In contrast to rimonabant, catecholaminergic stimulation (10 µM mazindol or 30 µM phentermine) increased spontaneous swimming without any effects on survival rate (data not shown), which corresponds to previous results seen in rodents [70], [71].

### Conclusion

Clinical appetite suppressants are associated with adverse effects due to inappropriate non-selective activity on signaling pathways, suggesting that drug targets of appetite regulation require further research. Both hyperphagia and anorexia are disorders related to dysregulation of appetite and urgently require treatment regimens without any adverse side effects. To address this problem, our fluorescence-based zebrafish feeding assay is a powerful tool in combination with MO knockdown or mutation screening. In summary, we have demonstrated a new high-throughput assay system for appetite-regulating drug screening and target gene discovery with high relevance to human diseases.
